# P-1868. The Impact of Location and ID Consultation on Outcomes in Gram-negative Bacteremia

**DOI:** 10.1093/ofid/ofaf695.2037

**Published:** 2026-01-11

**Authors:** David S Burgess, Emily Oliver, Katie B Olney, Donna R Burgess

**Affiliations:** University of Kentucky, Lexington, KY; UKHC, Lexington, Kentucky; University of Kentucky HealthCare, Lexington, KY; UK HealthCare, Lexington, KY

## Abstract

**Background:**

Gram-negative bacteremia remains a major cause of sepsis-related mortality. We aimed to identify key predictors of mortality using Classification and Regression Tree (CART) analysis in a large cohort of hospitalized patients with Gram-negative bacteremia.

**Methods:**

This retrospective study included adult patients (N=970) with Gram-negative bacteremia admitted to UK HealthCare from July 2022 to December 2024. Clinical, microbiologic, and outcomes data were analyzed. CART analysis identified predictors of in-hospital mortality stratified by ICU vs non-ICU status. ID consultation patterns were also examined.

**Results:**

Overall mortality was 15.8%. Median (IQR) age was 60 years (40, 70), with 53.2% male. Most infections were community-acquired (64.8%) and 42.8% of patients required ICU care. Median hospital LOS was 12 days (7, 26), ICU LOS 7.1 days (3.5, 13.8). Median weight was 78.6 kg (62.8, 98.4), and BMI 26.9 (22.2, 33.6). Comorbidities were common: CHF (33.4%), COPD (34.4%), diabetes without complications (37.4%), and MI (18.6%). Top pathogens included *E. coli* (40.1%), *K. pneumoniae* (15.1%), *P. aeruginosa* (9.4%), *S. marcescens* (6.4%), and *E. cloacae* (5.1%). Resistance genes were identified in 9.3% (90/970) of isolates, most commonly CTX-M (86/90, 95.6%), with rare occurrences of OXA, VIM, KPC, and NDM (1 each).

ID was consulted in 44.3% of cases, more frequently in non-ICU patients (49.6%) than ICU patients (37.3%) (p< 0.001). Consultation rates did not differ by infection source or severity (CCI, SOFA, or qPITT). CART analysis identified ICU admission as the primary predictor of mortality (ICU 26.4% vs non-ICU 7.8%). Among ICU patients, qPITT score was the next most influential factor (low: 17.4%, moderate: 27.1%, high: 427%). In non-ICU patients, hospital-acquired infections had higher mortality than community-acquired (16.2% vs 5.2%).

**Conclusion:**

In this large cohort, ICU admission and qPITT score were the strongest predictors of mortality, while community vs hospital acquisition differentiated risk among non-ICU patients. These findings support early severity scoring and consultation to guide therapy and improve outcomes in Gram-negative bacteremia.

**Disclosures:**

All Authors: No reported disclosures

